# Anticonvulsant Profile of Selected Medium-Chain Fatty Acids (MCFAs) Co-Administered with Metformin in Mice in Acute and Chronic Treatment

**DOI:** 10.3390/molecules28093810

**Published:** 2023-04-29

**Authors:** Mateusz Pieróg, Katarzyna Socała, Dorota Nieoczym, Elżbieta Wyska, Małgorzata Samorek-Pieróg, Piotr Wlaź

**Affiliations:** 1 Department of Animal Physiology and Pharmacology, Institute of Biological Sciences, Maria Curie-Skłodowska University, Akademicka 19, 20-033 Lublin, Poland; katarzyna.socala@mail.umcs.pl (K.S.); dorota.nieoczym@mail.umcs.pl (D.N.); piotr.wlaz@mail.umcs.pl (P.W.); 2 Department of Pharmacokinetics and Physical Pharmacy, Jagiellonian University Medical College, Medyczna 9, 30-688 Kraków, Poland; e.wyska@uj.edu.pl; 3 Department of Parasitology and Invasive Diseases, National Veterinary Research Institute, Partyzantów Avenue 57, 24-100 Puławy, Poland; malgorzata.samorek-pierog@piwet.pulawy.pl

**Keywords:** caproic acid, caprylic acid, lauric acid, metformin, mice, seizures, pentylenetetrazole, 6 Hz seizure test, MEST, seizure threshold

## Abstract

In contrast to the other components of the medium-chain triglycerides ketogenic diet (MCT KD), i.e., caprylic acid (CA8), a comprehensive evaluation of caproic (CA6) and lauric acids’ (CA12) properties in standard chemical and electrical seizure tests in mice has not yet been performed. We investigated their effects in maximal electroshock seizure threshold (MEST), 6 Hz seizure threshold and intravenous (i.v.) pentylenetetrazole (PTZ) seizure tests. Since ketone body production can be regulated by the activation of 5′AMP-activated protein kinase (AMPK), we hypothesized that metformin (an AMPK activator) enhance ketogenesis and would act synergistically with the fatty acids to inhibit convulsions. We assessed the effects of acute and chronic co-treatment with metformin and CA6/CA8 on seizures. CA6 and CA12 (p.o.) increased seizure threshold in the 6 Hz seizure test. CA6 at the highest tested dose (30 mmol/kg) developed toxicity in several mice, impaired motor performance and induced ketoacidosis. Acute and chronic co-treatment with metformin and CA6/CA8 did not affect seizure thresholds. Moreover, we observed the pro-convulsive effect of the acute co-administration of CA8 (5 mmol/kg) and metformin (100 mg/kg). Since this co-treatment was pro-convulsive, the safety profile and risk/benefit ratio of MCT KD and metformin concomitant therapy in epileptic patients should be further evaluated.

## 1. Introduction

Pharmacotherapy is the main form of epilepsy treatment, but about 30% of epileptic patients remain drug-resistant [1]. Considering that this chronic neurological disorder affects 65 million people worldwide [2] and the pathomechanism of the disease is not fully understood, the effective treatment of epilepsy in humans remains a problem. There is a need to find new antiseizure drugs or new therapeutic strategies for the epileptic disorder.

Numerous clinical studies have shown that a significant improvement in the health of some drug-resistant epileptic patients might be achieved with the use of the ketogenic diet (KD). Interestingly, a high-fat, low-carbohydrate and adequate-protein KD can be highly effective in suppressing seizures [3]. Classic KD with long-chain fatty acids/triglycerides, i.e., those with an aliphatic tail of more than 12 carbons (Wilder’s KD [4]), or a diet containing medium-chain fatty acids (MCFAs, 6–12 carbon atoms [5]), improve seizure control. The clinical efficacy of KDs cannot be simply explained. It is known that the body produces ketone bodies, which are the source of energy for the brain. Rodent studies have shown that some of the ketone bodies, especially acetone, have anticonvulsant properties [6,7,8,9,10]. Furthermore, the effect of the adenosine system on the efficacy of KD has also been postulated [11,12,13,14,15,16]. In contrast to long-chain fatty acids from the classical Wilder’s KD, the chemical properties of MCFAs, i.e., solubility in aqueous media, presence in an unbound fraction in the blood and affinity to carrier-mediated transporters, allows them to cross the blood–brain barrier (BBB) and exert direct and immediate pharmacological effects on the central nervous system [17]. MCT KD contains the following fatty acids: caprylic—CA8 (50–75% in the diet), capric—CA10 (23–45%), caproic—CA6 (1–3%) and lauric—CA12 (1–5%) [18,19]. Nakamura et al. [20] demonstrated that only a few fatty acids showed anticonvulsant efficacy against picrotoxin and/or pentylenetetrazole (PTZ)-induced convulsions in mice. On the other hand, later findings demonstrated the anticonvulsant efficacy of selected MCFAs in several other animal models of seizures, i.e., CA8 in the 6 Hz and the intravenous (i.v.) PTZ seizure tests in mice [21], nonanoic acid (CA9) in the rat status epilepticus model [22] and CA10 in the 6 Hz and maximal electroshock seizure threshold (MEST) tests in mice [17]. Although the anticonvulsant activity of CA8 and CA10 has been tested in standard chemical and electrical seizure tests in mice, such a comprehensive evaluation has not yet been performed for CA6 and CA12.

The endogenous production of ketone bodies is regulated, inter alia, by 5′AMP-activated protein kinase activity (AMPK). A potential drug that stimulates ketogenesis in astroglia by activating AMPK is metformin [23]. Metformin has been shown to control seizures in both acute and chronic models of epileptic seizures in rodents (e.g., PTZ, kainic acid or pilocarpine tests [24,25,26,27,28]), as well as in zebrafish [29]. Moreover, in clinical trials, metformin reduced the frequency of seizures in children and adults with tuberous sclerosis complex [30]. The mechanism of the anticonvulsant action of metformin may result not only from the activation of AMPK, but also from a reduction in oxidative damage of the brain [24], inhibition of the mammalian target of the rapamycin kinase (mTOR) pathway, the downregulation of brain-derived neurotrophic factor (BDNF) and Tropomyosin receptor kinase B (TrkB) expression [26], a reduction in α-synuclein synthesis [27] or a reduction in apoptosis [31]. More research is needed to explain its action in epilepsy. The clinical pharmacokinetics of metformin are well characterized in humans. In rodents, the drug administered orally (p.o.) in single or chronic doses crosses the BBB, and can be detected in the brain [32,33]. Based on the above information, we hypothesized that metformin might increase ketogenesis and act synergistically with MCFAs to inhibit convulsions.

The present study aimed to: (1) evaluate the potential anticonvulsant effects of two MCFAs—CA6 and CA12—as well as metformin in three acute seizure tests in mice, i.e., in the i.v. PTZ seizure test, MEST and 6 Hz-induced psychomotor seizure tests; (2) determine effects of acute and chronic co-treatment with metformin and two selected MCFAs on psychomotor seizure threshold in mice. Here, CA8—a previously studied fatty acid with antiepileptic properties [21]—was compared with the fatty acid assessed in this study—CA6. Additionally, some side effects of the studied MCFAs and metformin were studied, i.e., neuromuscular strength was evaluated using the grip-strength test and motor coordination was assessed in the chimney test. Metabolic modifications produced by the studied MCFAs and metformin were analyzed based on the changes in pH, glucose and β-hydroxybutyrate levels in blood.

## 2. Results

### 2.1. Effects of CA6 and CA12 in the MEST and 6 Hz-Induced Seizure Threshold Tests

**Figure 1:**
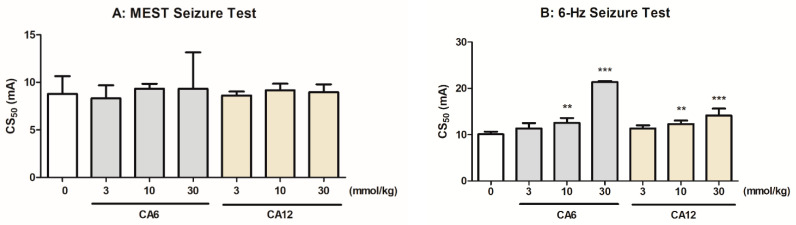
Effects of caproic acid (CA6) and lauric acid (CA12) on the seizure threshold in the maximal electroshock seizure threshold (MEST) (**A**) and 6 Hz-induced seizure threshold (**B**) tests in mice. Both CA6 and CA12 (doses on abscissa) were injected orally (p.o.), 30 min before the tests. Control animals received 0.5% methylcellulose. Data are expressed as CS_50_ (in mA) values with 95% confidence limits (*n* = 12–15 mice/dose). Each CS_50_ value represents the current intensity predicted to produce convulsions in 50% of mice tested. Statistical analysis was performed using one-way ANOVA followed by Tukey’s multiple comparison test. ** *p* < 0.01, *** *p* < 0.001 as compared to the control group.

The influences of CA6 and CA12 on the thresholds for the tonic hindlimb extension in the MEST test and for psychomotor seizures in the 6 Hz seizure threshold test are shown in Figure 1A and Figure 1B, respectively. CA6 and CA12 (3–30 mmol/kg, p.o.) had no significant effect on the current intensity necessary to induce tonic convulsions in the MEST test (one-way ANOVA, for CA6: F(3,23) = 0.2799, *p* = 0.8393; for CA12: F(3,24) = 0.2087, *p* = 0.8894; Figure 1A).

CA6 and CA12 dose-dependently increased the current intensity necessary to induce psychomotor seizures in the 6 Hz-induced seizure threshold test (one-way ANOVA, for CA6: F(3,20) = 42.99, *p* < 0.0001; for CA12: F(3,24) = 15.94, *p* < 0.0001). A post hoc analysis revealed that the threshold for 6 Hz-induced seizures was significantly elevated by both CA6 and CA12 at doses of 10 and 30 mmol/kg, p.o. (Figure 1B).

### 2.2. Effects of CA6 and CA12 on the Seizure Thresholds in the i.v. PTZ-Induced Seizure Test

**Figure 2:**
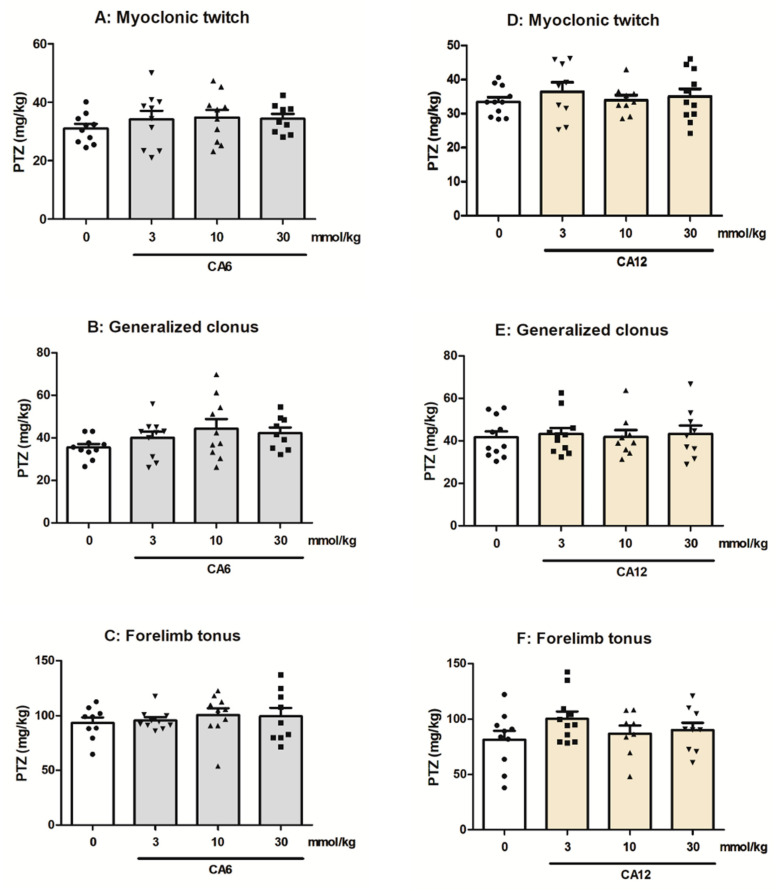
Effects of CA6 (**A**–**C**) and CA12 (**D**–**F**) on the seizure thresholds in the i.v. PTZ seizure test in mice. CA6 and CA12 (doses on abscissa) were administered 30 min prior to seizure testing; control animals received 0.5% methylcellulose. Each bar represents the mean dose (with individual measurements as symbols) and the standard error of the mean (SEM) of the PTZ (in mg/kg) necessary to produce myoclonic twitch (**A**,**D**), clonus (**B**,**E**) or tonus (**C**,**F**). Experimental groups consisted of 8–11 mice. Statistical analysis was performed using one-way ANOVA followed by Tukey’s multiple comparison test.

The effects of CA6 and CA12 on the seizure thresholds in the i.v. PTZ test are shown in Figure 2A–F. The studied MCFAs (3–30 mmol/kg, p.o.) had no significant effect on the threshold for PTZ-induced myoclonic twitch (one-way ANOVA, for CA6: F(3,38) = 0.5582, *p* = 0.6461 and for CA12: F(3,39) = 0.4215, *p* = 0.7387), generalized clonus (one-way ANOVA, for CA6: F(3,38) = 1.537, *p =* 0.222 and for CA12: F(3,39) = 0.0765, *p =* 0.9723) and forelimb tonus (one-way ANOVA, for CA6: F(3,37) = 0.3442, *p =* 0.7935 and for CA12: F(3,37) = 1.369, *p =* 0.2689).

### 2.3. Effects of CA6 and CA12 on Motor Coordination and Muscular Strength in Mice

Table 1 presents the influence of CA6 and CA12 at doses of 3, 10 and 30 mmol/kg on neuromuscular strength and motor coordination in mice. There were 30% impairments of motor coordination in the chimney test after CA6 administration at the dose of 30 mmol/kg (Fisher’s exact test: *p* > 0.05). Moreover, CA6 at the same dose tested had effects on muscular strength, as assessed by the grip-strength test (one-way ANOVA: F(3,36) = 6.059, *p* = 0.0019). Any doses of CA12 did not significantly impair grip-strength or motor coordination in mice (one-way ANOVA: F(3,36) = 1.851, *p* = 0.1555).

### 2.4. Effects of CA6 and CA12 on the Blood pH and Concentrations of Glucose and β-Hydroxybutyrate

**Figure 3:**
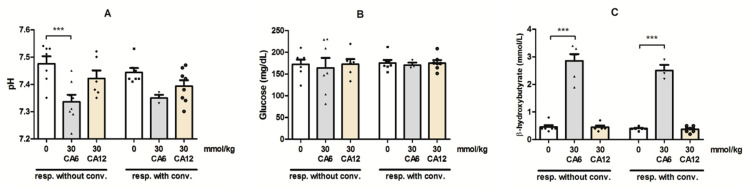
Changes in blood pH (**A**), blood glucose concentration (**B**) and β-hydroxybutyrate concentration (**C**) in mice after treatment with CA6 and CA12. The effects of CA6 (30 mmol/kg) and CA12 (30 mmol/kg) administration (p.o.) on blood biochemical parameters were investigated immediately after the 6 Hz seizure test. The doses are shown on the abscissa. Experimental groups consisted of 3–8 animals. Data are presented as means (with individual measurements as symbols) and SEM for groups that did or did not respond with post-stimulus convulsions. Statistical analysis was performed using two-way ANOVA followed by Bonferroni multiple comparison test. *** *p* < 0.001 as compared to the control group.

The changes in blood pH, glucose and β-hydroxybutyrate concentrations in mice treated with CA6/CA12 are presented in Figure 3A–C.

A significant effect on trunk blood pH in mice was observed after the administration of both MCFAs (Figure 3A). Two-way ANOVA demonstrated that there was a significant effect of the MCFAs treatment (F(2,33) = 10.08, *p* = 0.0004), but no effect of the response to stimuli on pH (F(1,33) = 0.52, *p* = 0.4772) and no effect of the response to stimuli × MCFAs treatment interaction (F(2, 33) = 0.4760, *p* = 0.6255). The Bonferroni post hoc test showed that the administration of MCFAs did not significantly change the pH in the group that responded without convulsions when compared to the group that responded with convulsions. On the other hand, CA6 at the dose of 30 mmol/kg decreased the pH in the group that responded without convulsions when compared to the control group.

Two-way ANOVA demonstrated that there was no effect of the response to stimuli (F(1,31) = 0.1249, *p* = 0.7261), no effect of the MCFAs treatment (F(2,31) = 0.1485, *p* = 0.8626) and no effect of the response to stimuli × MCFAs treatment interaction (F(2, 31) = 0.0122, *p* = 0.9879) regarding the glucose concentration in mice blood (Figure 3B).

There was significant effect of the MCFAs treatment on the β-hydroxybutyrate concentration (two-way ANOVA: F(2,27) = 193.6, *p* < 0.0001), but no significant effect of the response to stimuli (F(1,27) = 2.217, *p* = 0.1481) and no significant response to stimuli × MCFAs treatment interaction (F(2,27) = 0.7699, *p* = 0.4730). The Bonferroni *post hoc* test showed that the administration of MCFAs did not significantly change the β-hydroxybutyrate concentration in the group that responded without convulsions when compared to the group that responded with convulsions. At the tested dose, 30 mmol/kg, only CA6 increased the β-hydroxybutyrate concentration in the groups that responded with and without convulsions when compared to the control group (Figure 3C).

### 2.5. Effects of Metformin on the Seizure Threshold in the MEST and 6 Hz-Iduced Seizure Tests

**Figure 4:**
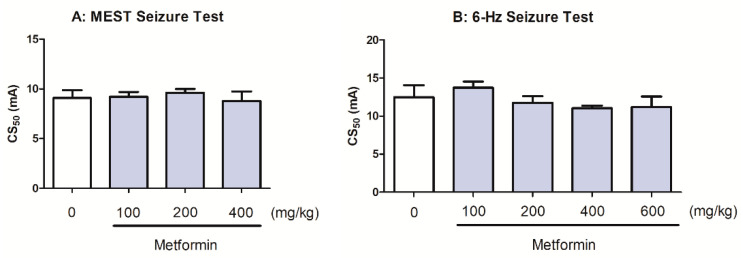
Effect of metformin on the seizure threshold in the MEST (**A**) and 6 Hz-induced seizure (**B**) tests in mice. Metformin (doses on abscissa) was injected p.o. 60 min before the tests. Control animals received saline. Data are expressed as CS_50_ (in mA) values with 95% confidence limits (*n* = 20 mice/group). Each CS_50_ value represents the current intensity predicted to produce convulsions in 50% of mice tested. Statistical analysis was performed using one-way ANOVA followed by Tukey’s multiple comparison test.

The effect of metformin on the seizure thresholds in the MEST and 6 Hz-induced seizure tests is presented in Figure 4. Metformin (at a dose range 100–400 mg/kg) did not have a significant effect on the current intensity necessary to induce tonic convulsions in the MEST seizure test (one-way ANOVA: F(3,34) = 1.024, *p* = 0.3941; Figure 4A). Similarly, metformin (at a dose range 100–600 mg/kg) had no significant effect on the psychomotor seizure threshold in the 6 Hz-induced seizure threshold test (one-way ANOVA: F(4,42) = 4.453, *p* = 0.0043; Figure 4B).

### 2.6. The Effect of Metformin in the i.v. PTZ-Induced Seizure Threshold Test

**Figure 5:**
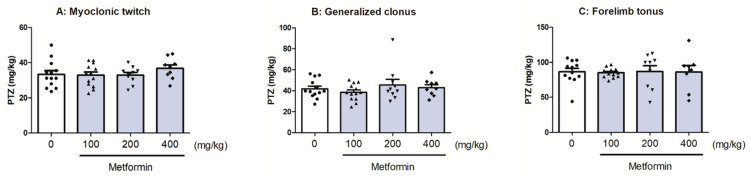
Effect of metformin on seizure thresholds in the i.v. PTZ seizure test. Metformin (doses on abscissa) was administered 60 min (p.o.) prior to seizure test; control animals received saline injections. Each bar represents the mean dose (with individual measurements as symbols) and SEM of the PTZ (in mg/kg) necessary to produce myoclonic twitch (**A**), clonus (**B**), or tonus (**C**). Experimental groups consisted of 8–13 mice. Statistical analysis was performed using one-way ANOVA followed by Tukey’s multiple comparison test.

The effects of metformin on the seizure thresholds in the i.v. PTZ test are shown in Figure 5. Metformin (at doses ranging from 100 to 400 mg/kg) had no significant effect on the threshold for PTZ-induced myoclonic twitch (one-way ANOVA: F(3,41) = 0.819, *p* = 0.4909), generalized clonus (one-way ANOVA: F(3,41) = 0.857, *p* = 0.4711) or forelimb tonus (one-way ANOVA: F(3,37) = 0.019, *p* = 0.9964).

### 2.7. Effects of Metformin on Motor Coordination and Muscular Strength in Mice

Metformin (100–600 mg/kg) did not significantly influence motor coordination in mice. As shown in Table 2, metformin also did not significantly change the muscle strength in mice (one-way ANOVA: F(4,45) = 1.777, *p* = 0.1500).

### 2.8. Effects of Acute and Chronic Treatment with Metformin, CA6, CA8 and Their Combination in the 6 Hz-Induced Seizure Test

**Figure 6:**
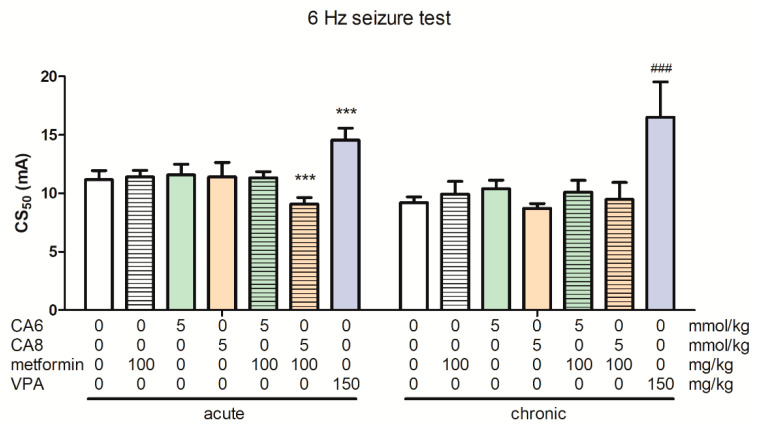
Effects of acute and chronic (14 days) treatment with VPA (150 mg/kg) and co-treatment with metformin (100 mg/kg) and CA6 (5 mmol/kg)/CA8 (5 mmol/kg) on the 6 Hz-induced seizure thresholds. Metformin was administered 60 min (p.o.), CA6, CA8 (p.o.) and VPA (intraperitoneally, i.p.) 30 min before the seizure test. The doses are shown on the abscissa. Each experimental group consisted of 15–20 animals. The unequal group sizes result from post-administration complications and mortality during chronic treatment. Data are presented as CS_50_ (in mA) values with upper 95% confidence limits. Each CS_50_ value represents the current intensity predicted to produce convulsions in 50% of mice tested. Statistical analysis was performed using one-way ANOVA followed by Tukey’s multiple comparison test. *** *p* < 0.001 as compared to the acute-treated control group; ### *p* < 0.001 as compared to the chronic-treated control group.

The effects of acute and chronic treatment with metformin, CA6, CA8 and their combination on the psychomotor seizure threshold are shown in Figure 6 (one-way ANOVA: F(6,59) = 16.10, *p* < 0.0001 for acute-treated groups and F(6,55) = 16.76, *p* < 0.0001 for chronic-treated groups).

The acute co-administration of CA8 (5 mmol/kg) and metformin (100 mg/kg) significantly decreased the CS_50_ when compared to the control group. Positive control (VPA at 150 mg/kg) significantly increased the seizure threshold after acute and chronic treatment (*p* < 0.001 vs. the respective vehicle-treated group).

### 2.9. Effects of Acute and Chronic Co-Treatment with Metformin and CA6/CA8 on Motor Coordination and Muscular Strength in Mice

As shown in Table 3, neither acute nor chronic treatment with metformin, CA6, CA8 or their combinations significantly changed grip-strength or motor coordination in mice. 

### 2.10. Effects of Chronic Treatment with Metformin, CA6, CA8 and Their Combinations on Body Weight

As shown in Table 4, chronic treatment with CA6 or its combination with metformin resulted in the death of several mice, but the mortality was not statistically significant (the incidence of death was 2/30 and 8/30, respectively). The unequal group sizes at the end of the paradigm were not due to the toxicity of the substances (due to their low doses), but rather to post-administration complications during chronic treatment, e.g., accidental aspiration and choking, or esophagus injures and inflammation.

**Figure 7:**
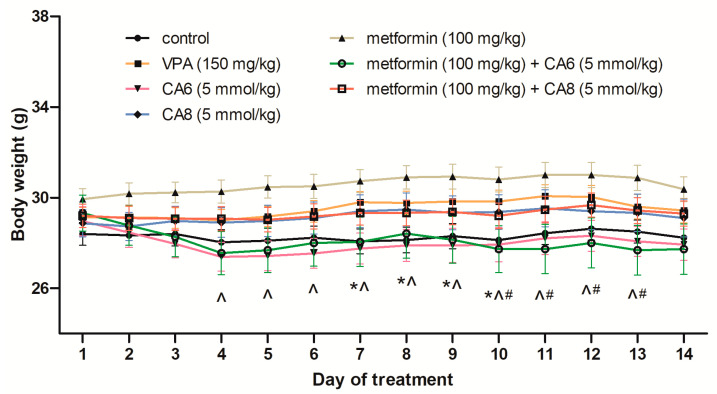
Effects of chronic treatment with metformin (100 mg/kg), CA6 (5 mmol/kg), CA8 (5 mmol/kg) and their combination as well as VPA (150 mg/kg) on the body weight in mice. Substances/vehicles were injected p.o. or i.p. (only VPA) once daily on every day. Data are presented as means and SEM of weight (in g), *n* = 22–30. The unequal group sizes result from post-administration complications and mortality during chronic treatment. Statistical analysis was performed using two-way ANOVA followed by Tukey’s multiple comparison test. * *p* < 0.05 when comparing the metformin-treated group to the control group. ^ *p* < 0.05 when comparing the metformin-treated group to the CA6-treated group; # *p* < 0.05 when comparing the metformin-treated group to the metformin + CA6-treated group.

We assessed the body weight of mice subjected to the chronic treatment with metformin, CA6, CA8 and their combination (Figure 7). We saw significant effects of the MCFAs treatment on body weight (two-way ANOVA: F(6,2702) = 29.93; *p* < 0.0001), but no significant effect of the time (two-way ANOVA: F(13,2702) = 0.6349; *p* = 0.8264) and no significant treatment × time interaction (two-way ANOVA: F(78,2702) = 0.2057; *p* > 0.9999). The Tukey’s post hoc test showed that metformin significantly increased the body weight in mice as compared to the control group (*p* < 0.05 from day 7 to day 10 of treatment, respectively). On the other hand, CA6 significantly decreased the body weight in mice as compared to the metformin-treated group (*p* < 0.05 from day 4 to day 13 of treatment, respectively). The co-administration of metformin with CA6 also decreased the body weight in mice as compared to the metformin-treated group (*p* < 0.05 from day 10 to day 13 of treatment, respectively).

### 2.11. Effects of Chronic Treatment with Metformin, CA6, CA8 and Their Combinations on the Blood pH and Concentrations of Glucose and β-Hydroxybutyrate

**Figure 8:**
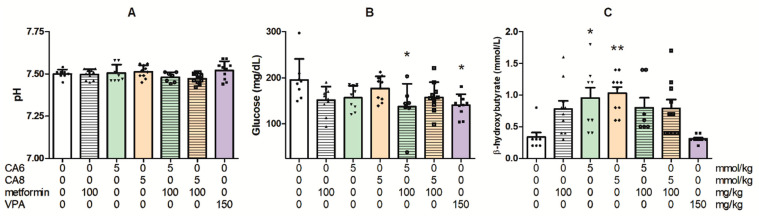
Changes in trunk blood pH (**A**), glucose concentration (**B**) and β-hydroxybutyrate concentration (**C**) in non-stimulated (sham) mice after chronic treatment with metformin (100 mg/kg), CA6 (5 mmol/kg)/CA8 (5mmol/kg) or their combinations, as well as VPA (150 mg/kg). The blood biochemical parameters were investigated 60 min after the last treatment with metformin and 30 min after the last CA6, CA8 and VPA injection. The doses are shown on the abscissa. Experimental groups consisted of 7–10 animals. Unequal group sizes result from post-administration complications and mortality during chronic treatment. Data are presented as means (with individual measurements as symbols) and SEM of trunk blood pH, glucose concentration (in mg/dL) or β-hydroxybutyrate concentration (in mmol/L). Statistical analysis was performed using one-way ANOVA followed by Tukey’s multiple comparison test. * *p* < 0.05, ** *p* < 0.01 as compared to the control group.

The changes in trunk blood pH, glucose and β-hydroxybutyrate concentrations after chronic treatment (14 days) with metformin, CA6, CA8 or their combinations in non-stimulated mice are presented in Figure 8.

The chronic administration of metformin as well as both tested MCFAs did not influence blood pH (one-way ANOVA: F(6,57) = 1.88, *p* = 0.0997; Figure 8A).

One-way ANOVA demonstrated that there was a significant effect of co-treatment with CA6 and metformin on the glucose concentration in mice blood (F(6,57) = 3.134, *p* = 0.01; Figure 8B).

MCFAs treatment significantly increased the β-hydroxybutyrate concentration in the mouse blood when compared to the control group (one-way ANOVA: F(6,57) = 5.799, *p* < 0.0001; Figure 8C).

## 3. Discussion

MCFAs can serve as metabolic substrates for the production of ketone bodies during MCT KD exposure [7]. The present study adds to the evidence that both CA6 and CA12 may have acute anticonvulsant properties in relation to the acute seizure threshold test in mice. After the first phase of the experiment, we assumed that not only the main fatty acids, i.e., CA8 and CA10 [17,21], but also the secondary components of MCT KD, like CA6 and CA12, may contribute to the overall efficacy of this diet in epilepsy patients.

Pretreatment times for CA6 and CA12 (30 min) were based on those reported for other MCFAs in the literature [17,21,34]. CA6 and CA12 at the doses of 10 and 30 mmol/kg showed an acute anticonvulsant efficacy in the 6 Hz seizure threshold test. However, both MCFAs had no significant effect in the i.v. PTZ and MEST tests. The obtained results are predominantly in agreement with those observed for MCFAs (with an aliphatic tail 6–12 carbons long). Keane et al. [35] and Chapman et al. [36] showed that CA6 at doses of 0.5–4 mmol/kg (i.p.) had no antiseizure activity against i.p. PTZ-induced seizures or audiogenic seizures in mice. Furthermore, CA6 did not significantly alter the brain GABA level. In one study by Nakamura et al. [20], CA6, CA12 and other carboxylic acids were administered i.p. at two doses 0.5 and 1 mmol/kg in mice 15 min before subcutaneous injections of either picrotoxin and PTZ. They showed that the onsets of the clonic convulsions induced by PTZ and picrotoxin were delayed only by CA12 (1 mmol/kg). It also increased the survival to lethality induced by both proconvulsants. The other MCFA, CA8 (20 and 30 mmol/kg, p.o.), increased the threshold for i.v. PTZ-induced myoclonic and clonic convulsions, but not tonic convulsions in mice. It also increased the threshold for 6 Hz psychomotor seizures (10–30 mmol/kg), but was ineffective in the MEST test [21]. Another fatty acid, CA10, showed significant anticonvulsant properties by increasing seizure thresholds in the 6 Hz (10 and 30 mmol/kg, p.o.) and MEST seizure tests (50 mmol/kg); however, it was ineffective in the i.v. PTZ seizure test [17]. The present study is in line with the abovementioned reports—selected fatty acids at similar doses were equally efficacious and potent in the 6 Hz seizure test. However, they have different anticonvulsant profiles in the MEST test or i.v. PTZ seizure model, suggesting that they are not entirely similar in terms of pharmacological efficacy. Similarly, the KD protects against epileptic seizures in the 6 Hz rodent model, but is weakly active in the mouse MES and inactive in the rat MES, or has no effect on PTZ-induced seizures [37,38]. Surprisingly, ketonic diet-fed mice were even more susceptible to seizures induced by the MEST seizure test [39].

There were no significant changes in glucose, pH or β-hydroxybutyrate level after the CA12 treatment. CA6 at a dose of 30 mmol/kg had no effect on glucose level, but it significantly decreased pH and increased the concentration of β-hydroxybutyrate in the trunk blood. Interestingly, we observed signs of acute toxicity in relation to the highest dose of CA6, including sedation, blood in urine as well as the death of mice. Three mice out of forty-five injected with 30 mmol/kg CA6 (p.o.) died within 60 min of administration, blood in urine was observed in less than ten mice, and sedation was observed in all subjects. In contrast to rats, there is no information in the literature on the LD_50_ of CA6 after oral administration to mice. Since the acute oral LD_50_ value in male rats is 6440 mg/kg [40], it can be concluded that the applied dose of CA6 (30 mmol/kg = 3484.8 mg/kg) may lead to systemic toxic effects in the mice tested. Changes in biochemical studies show that the highest dose of CA6 caused diabetic ketosis and acidosis in mice with the following detrimental effects on neuromuscular strength and motor coordination in mice. It is possible that the in vivo anticonvulsant effect of CA6 was due to acidosis and the involvement of the acid sensing ion channel 1a (ASIC1a), which regulates neuron excitability [41]. In diabetic ketoacidosis, significant increases in blood ketone and glucose levels are accompanied by a lowered blood pH [42]. Interestingly, in the Socala et al. [34] study, ketosis, but no acidosis, was observed in both fasted and non-fasted mice treated with CA8, and hyperglycemia in the fasted group was not observed. The results on the blood biochemical parameters reported in our study are consistent with previous findings by Wlaź et al. [17,21]. In the abovementioned studies, the decreased blood pH is a consequence of acute CA8 or CA10 administration and ketosis, but not of hyperglycemia, suggesting no metabolic interactions between glucose and ketone bodies. Furthermore, Hartman et al. [38] reported that both the KD and calorie restrictions decreased blood glucose levels in juvenile mice, but only the KD significantly elevated the threshold for 6 Hz-induced seizures. According to these findings, the anticonvulsant mechanisms that underlie calorie restriction and the KD appear to be distinct in mice. It is noteworthy that changes in blood glucose levels do not correlate with the effectiveness of the KD in children consuming KD for seizure control [43]. To sum up, mid to high doses of CA6 and CA12 may have acute anticonvulsant effects in the 6 Hz seizure test, but should be used under controlled conditions to avoid unexpected toxicity in mice.

The overall results of the present study show that, in contrast to CA6, there was no relationship between the level of ketosis and CA12 anticonvulsant efficacy in the 6 Hz seizure test. Consistent with the other rodent studies, elevated β-hydroxybutyrate is not a reliable biomarker for effective seizure control [44,45,46,47]. However, in the body, β-hydroxybutyrate is converted into acetoacetate and acetone. Given that both products have anticonvulsant properties [6,7,8,9], the involvement of these metabolites in the anticonvulsant activity produced by CA6 and CA12 cannot be ruled out.

Many studies have shown that metformin has neuroprotective properties (i.e., [29,48]). Several studies also suggest that it may regulate seizure progression in animal seizure models [24,28]. It is well documented that metformin can activate AMPK signaling, an important energy balance sensor [49,50]. According to Yang et al.’s [25] findings, since the AMPK expression level was decreased in the epileptic mouse brain, which had chronic and acute seizures, treatment with metformin upregulated the p-AMPK level (phosphorylated and activated form), and may play a role in seizure control. Interestingly, some in vivo and in vitro studies have suggested that metformin may promote the acidification of neurons by activating AMPK and blocking aerobic respiration [51,52]. Acidification may be a potential mechanism by which metformin can promote seizure termination [25,41]. Although we did not measure extracellular brain pH, the biochemical parameters of the trunk blood ruled out ketoacidosis and lactic acidosis in mice treated for 14 days with 100 mg/kg of metformin (p.o.). Moreover, our study did not confirm the acute (treatment with a dose range of 100–600 mg/kg, p.o.) and chronic antiseizure effects of metformin in different acute seizure models. Although the mechanism responsible for the anticonvulsant activity of metformin is unknown, its lack of effect on three seizures models makes the theory of AMPK activation followed by acidification as contributing to the anticonvulsant activity in vivo unlikely. In general, chronic metformin treatment (administered in a dose range of 100–250 mg/kg per day or in drinking water, i.e., 2 mg/mL) has pronounced antiepileptic potential in chemically and electrically induced chronic seizure models in rodents, i.e., via delaying the onset of seizure, reducing seizure frequency and duration [28]. The abovementioned behavioral effects were not observed in acute seizure models. In Brueggeman et al.’s study [29], metformin suppresses acute PTZ seizures in zebrafish without repressing the motility or activity of the central nervous system. As shown in our study, acute or chronic metformin treatment did not modify the seizure threshold in acute i.v. PTZ, MEST or 6 Hz seizure models. The obtained results are in agreement with those observed by Yang et al. in the i.p. PTZ model (seizures were induced by injection of 70 mg/kg PTZ) [25]. They showed that metformin treatment does not alter the seizure severity that is assessed by either the maximum Racine score or the incidence of generalized tonic-clonic seizures in this model. Therefore, they suggest that chronic metformin treatment regulates PTZ-induced acute seizures through facilitating their termination, but not affecting their initiation.

Metformin was found to activate AMPK and induce increases in ketone bodies production in astroglia [23,53]. In recent years, non-pharmacological treatment methods of drug-resistant epilepsy have been increasingly considered [3,54]. Among them, KD with MCTs has aroused interest in understanding the mechanisms responsible for the anticonvulsant and possibly antiepileptic properties of KD [22]. The above facts prompted us to assess the hypothesis of whether metformin increased ketogenesis and would act synergistically with selected MCFAs to inhibit convulsions in animals. The analysis of the results of the 6 Hz seizure test and the biochemical analysis of the trunk blood determined the choice of CA6 over CA12 for this part of the experiment. The dose of CA6 depended on the results of our previous procedures; in the case of CA8, it was based on data from the literature [21]. Treatment with metformin, CA6 and CA8 is used to protect animals from possible side effects (i.e., toxicity after addition of keto- and lactic acidosis, rapid weight loss); therefore, we chose the subeffective doses of CA6 and CA8 administered concomitantly with a non-effective dose of metformin. Biochemical studies have revealed a statistically significant increase in ketone bodies’ levels in mice treated with CA6 and CA8 as compared to the control group, and a non-significant increase in ketone bodies levels in mice treated simultaneously with metformin and CA6 or CA8. However, in the present study, the use of a combination of substances did not allow the observation of an anticonvulsant effect in 6 Hz seizure test.

As shown here, combining metformin with a subeffective dose of CA8 decreased the seizure threshold in the 6 Hz test after acute treatment. In the context of the previous research findings for CA8 [21,34], this result is dissimilar. The mechanisms causing these changes in seizure threshold also remain unclear. In vivo, severe hypoglycemia is often associated with seizures [55], but in our study, selective or concomitant chronic treatment with low doses of CA8 and metformin did not alter glucose level. On the other hand, low glucose levels are essential to maintain seizure suppression in animals and KD patients [56,57]. Hypoglycemia cannot explain the proconvulsant effects observed when testing the metformin + CA8 group. We did not observe the development of ketosis (as measured by blood level of β-hydroxybutyrate) or acidosis in mice treated for 2 weeks with CA8 and metformin. We also did not observe any deaths following their administration. Paradoxically, the treatment of mice with a combination of CA6 and metformin resulted in hypoglycemia, elevated β-hydroxybutyrate concentration in blood and a life-threatening weight loss, but with no pro-convulsant effect. It seems that the reduction in seizure threshold in the 6 Hz test after co-treatment with metformin and CA8 was not associated with weight gain, ketosis, acidosis or hypoglycemia. In a high-fat diet, many variables influence seizures, and we still cannot determine how each of these variables accounts for the differences in seizure severity/protection observed in several experimental groups. Some studies revealed mixed results (i.e., ranging from no effect to anticonvulsant or proconvulsant) in terms of the effects of the KDs on acute mouse seizures models [37,39,44,46,58,59,60]. In Samala et al.’s study [61], they showed that KD’s anticonvulsant effect was limited to the 6 Hz model, required chronic feeding with 6:1 fat content, and was independent from lowering plasma glucose. Here, the observed that the acute increase in seizure tendency induced by CA8, the major component of KD MCT, co-administered with metformin was not due to any particular mechanism, but was instead more likely to represent a non-specific brain change.

## 4. Materials and Methods

### 4.1. Animals and Experiment Design

Adult male Albino Swiss mice weighing 25–35 g obtained from a licensed breeder (Laboratory Animals Breeding, Warsaw, Poland) were used. The total number of animals used in the present study was 920. The animals were housed in Makrolon cages (37 cm × 21 cm × 14 cm) in groups of 8–9/cage. Laboratory conditions were strictly controlled as follows: temperature maintained at 21–24 °C, relative humidity at 45–65%, an artificial 12/12 h light/dark regime (light on at 6:00 a.m.), and free access to chow pellets (Agropol S.J., Motycz, Poland) and tap water. The mice were used after at least one week of acclimatization. All behavioral experiments were performed at the same time of day, between 8:00 a.m. and 3:00 p.m., after a minimum 30 min adaptation period to the conditions kept in the experimental room. The animals were habituated to handling for one week prior to behavioral assays to minimize stress and its effects on experimental variability. Only male mice were used in the study to rule out the potential effect of the estrous cycle on seizure susceptibility [62].

All behavioral tests were performed by experimenters blinded to the treatment. The animals were assigned to the experimental groups as follows:

- Procedures with CA6 and CA12: 15 animals/group in the i.v. PTZ test, 15 animals/group in the MEST test, 15 animals/group in the 6 Hz-induced psychomotor seizure test, and 10 animals/group in the grip-strength test and the chimney test. Before the 6 Hz seizure test, three mice died after treatment with 30 mmol/kg CA6 and were excluded from the analysis, as well as one mouse administered with 30 mmol/kg of CA12;
- Procedures with metformin: 15 animals/group in the i.v. PTZ test, 20 animals/group in the MEST test, 20 animals/group in the 6 Hz-induced psychomotor seizure test, and 10 animals/group in the grip-strength test and the chimney test;
- Procedures with a combination of metformin and selected MCFAs (CA6 and CA8) after acute and chronic treatment:
  (1) Acute effects—20 animals/group in the 6 Hz-induced psychomotor seizure test, and 10 animals/group in the grip-strength test and the chimney test. In all groups, vehicles were injected repeatedly every 24 h for 13 consecutive days. The acute effect of substances on the seizure threshold was assessed on the following (14th) day;
  (2) Chronic effects—20 animals/group in the 6 Hz-induced psychomotor seizure test, 10 animals/group in the grip-strength test and the chimney test, and 10 animals/group were used to measure the glucose level, acidosis and ketosis. In all groups, substances and vehicles were injected repeatedly every 24 h for 14 consecutive days. The chronic effect of substances on the seizure threshold was assessed on the last day of treatment (after 14 injections).

In order to compare the effects of substances after acute and chronic administration, as well as to eliminate other variables, animals from both procedures were treated identically. In the case of testing the anticonvulsant activity of a substance, it is of particular importance to determine whether the effects of their action after a single administration will be similar to those observed after repeated administration.

Since the grip-strength test and the chimney test are very quick and non-invasive procedures, these tests were performed in the same groups of animals shortly before the seizure tests. The effects of MCFAs administration on biochemical parameters of trunk blood were investigated immediately after the 6 Hz seizure test. This allowed us to limit the total number of animals used in the study, as was in accordance with the guidelines of the Ethics Committee. Biochemical parameters of trunk blood after MCFAs and metformin co-administration were measured in non-stimulated (sham) animals—mice received the same doses of substances or vehicles, but they did not receive the electrical stimulus.

All procedures were conducted in accordance with the European Union Directive of 22 September 2010 (2010/63/EU) and Polish legislation acts concerning animal experimentations. The experimental procedures and protocols were approved by the Local Ethics Committee in Lublin (license no 44/2021 and 50/2022). The experiment has been designed to effectively implement the principles of replacement, reduction and refinement (3Rs principle). The number of animals in each experimental group was reduced to the minimum needed to achieve consistent and reproducible results and their reliable statistical analysis. All efforts were made to minimize animal suffering in the study.

### 4.2. Drugs

Caproic acid (CA6, MW = 116.16 g; also called hexanoic acid), caprylic acid (CA8, MW = 144.21 g; also called octanoic acid) and lauric acid (CA12, MW = 200.32 g; also called dodecanoic acid) were suspended in a 0.5% aqueous solution of methyl cellulose (all from Sigma-Aldrich, St. Louis, MO, USA). Metformin (Auro Laboratories Limited, Mumbai, India) was dissolved in saline (0.9% NaCl). Doses of CA6, CA8 and CA12 were expressed in mmol/kg, and doses of metformin in mg/kg. MCFAs and metformin were administered p.o. by gastric gavage in a volume of 10 mL/kg of body weight before the respective test. The pretreatment time (30 min) for all MCFAs was based on those reported for other MCFAs in the literature [17,21,34]. The pretreatment time with metformin in mice (60 min) was also based on that reported previously [32,33]. All suspensions/solutions were prepared freshly. The negative control groups received respective vehicles. The positive control group received valproate (VPA, sodium salt, Sigma-Aldrich, St. Louis, MO, USA), which was administered i.p., 30 min before the tests. PTZ (Sigma-Aldrich, Poznań, Poland) was dissolved in saline and infused i.v.

### 4.3. I.v. PTZ Seizure Threshold Test

The i.v. PTZ seizure threshold test was performed as described before [21]. PTZ was administered to the unrestrained animals at a constant rate of 0.2 mL/min. During the test, mice were observed by a trained observer for the occurrence of three types of seizures, i.e., (1) the initial myoclonic twitch, (2) generalized clonus with loss of righting reflex and (3) tonic forelimb extension. The time between the start of the infusion and the onset of each of these endpoints was recorded and used to calculate seizure thresholds for the respective types of seizures. The seizure thresholds were calculated according to the formula:

$$\mathrm{PTZ}\;\left(\frac{\mathrm{mg}}{\mathrm{kg}}\right)=\frac{\mathrm{infusion}\;\mathrm{duration}\;\left(s\right)\;\times \;\mathrm{infusion}\;\mathrm{rate}\;\left(\frac{\mathrm{mL}}{s}\right)\;\times \;\mathrm{PTZ}\;\mathrm{concentration}\;\left(\frac{\mathrm{mg}}{\mathrm{mL}}\right)}{\mathrm{body}\;\mathrm{weight}\;\left(\mathrm{kg}\right)}$$

Data are expressed as the mean amount of PTZ (mg/kg) and SEM. Tonic convulsions were usually lethal for mice, otherwise all surviving animals were euthanized immediately after the end of the infusion.

### 4.4. Maximal Electroconvulsions

Generalized tonic-clonic convulsions were induced by constant current stimuli (50 Hz sine-wave, 0.2 s) applied via saline-soaked transcorneal electrodes. The current was delivered after the application of ocular anesthetic (1% solution of tetracaine hydrochloride applied into each eye 1 min before the stimulation) with the usage of a rodent shocker (type 221; Hugo Sachs Elektronik, Freiburg, Germany). During stimulation, mice were restrained manually and immediately after stimulation were placed in a transparent box and observed for the presence or absence of seizure activity. The criterion for the occurrence of seizure activity was tonic hind limb extension (also called tonus), manifesting as the rigid extension of the hind limbs that exceeds a 90° angle with the body. The thresholds for maximal electroconvulsions were assessed by the “up-and-down” method as described by Kimball et al. [63]. Each mouse was stimulated only once at any given current intensity. The data obtained in the groups were used to determine the threshold current causing endpoint in 50% of mice (CS_50_ with confidence limits for 95% probability).

### 4.5. Psychomotor Seizure Threshold Test

Psychomotor seizures were induced via corneal stimulation. Square-wave alternating current stimuli (0.2 ms duration pulses at 6 Hz for 3 s) were applied via saline-soaked corneal electrodes using a Grass S48 stimulator coupled with a constant current unit CCU1 (both from Grass Technologies, West Warwick, RI, USA). A drop of ocular anesthetic (1% solution of tetracaine hydrochloride) was placed on the corneas before the stimulation and the electrodes were soaked in the normal saline immediately before testing to ensure a good electrical contact. Mice were restrained manually during electrical stimulation and immediately following stimulation were placed in a Plexiglas arena (37 cm × 21 cm × 14 cm) for behavioral observation. Psychomotor seizures were characterized by immobility or stun posture, jaw and forelimb clonus, twitching of the vibrissae and elevated or Straub tail [64]. The lack of the features listed above or the resumption of normal exploratory behavior within 10 s after stimulation were considered as absence of seizures. The mice were subjected to stimuli with different current intensities according to an “up-and-down” method [63]. Each animal was stimulated only once at any given current intensity that was lowered or raised by 0.06-log intervals depending on whether the previously stimulated animal did or did not respond with convulsions, respectively. The data obtained in the groups were used to determine the threshold current intensity that cause psychomotor seizures in 50% of mice tested (CS_50_ with confidence limits for 95% probability).

### 4.6. Behavioral Effects in Grip-Strength Tests

The grip-strength apparatus (BioSeb, Chaville, France) consisted of a steel wire grid (8 cm × 8 cm) connected to an isometric force transducer. Animals were lifted by their tails so that they could grasp the grid with their forepaws. The mice were then gently pulled backward until they released the grid. The maximal force in newtons (N) exerted by the mouse before losing grip was measured. The procedure was repeated three times and the mean force exerted by each mouse before losing grip was recorded. Since body weight affects the grip force, the mean force was normalized to body weight and expressed in mN/g and SEM.

### 4.7. Behavioral Effects in Chimney Test

During the chimney test mice, had to climb backwards up the plastic transparent cylinder (inner diameter 3 cm and length 30 cm) with a gravelly inside. The inability of mice to escape from the cylinder within 60 s was considered as a motor impairment [65]. The results obtained in the test were presented as percent of impaired mice in each group.

### 4.8. Determination of Blood pH and Concentrations of Glucose and β-Hydroxybutyrate

Blood pH as well as glucose (mg/dL) and β-hydroxybutyrate (mmol/L) concentrations were measured immediately after decapitation in the trunk blood at a pretreatment time consistent with that of the seizure tests. Blood samples were collected into Eppendorf tubes and blood pH was measured using a CPC-551 pH meter (Elmetron, Poland). Concentrations of glucose and β-hydroxybutyrate were measured using a test strip system and reader (Optium Xido blood glucose meter with Optium Xido blood ketone test strips and blood glucose test strips; Abbott Diabetes Care Ltd., Range Road Witney, Oxon, UK), as described elsewhere [17,21]. Values were expressed as group means and SEM.

### 4.9. Statistical Analysis

Data were analyzed by one-way analysis of variance (one-way ANOVA) followed by the Tukey’s post hoc test for multiple comparison. The results from the chimney test and mice mortality were analyzed with the Fisher’s exact probability test. Blood biochemical parameters (pH, glucose and β-hydroxybutyrate concentrations) were analyzed by one-way ANOVA followed by the Tukey’s post hoc test or two-way ANOVA followed by the Bonferroni post hoc test. Data reflecting the effects of chronic treatment with substances on body weight in mice were analyzed with two-way ANOVA followed by Tukey’s multiple comparison test. The statistical analysis was carried out using GraphPad Prism for Windows version 5.03 (GraphPad Software, San Diego, CA, USA). *p* < 0.05 was considered statistically significant. No statistical method was used to predetermine sample size.

## 5. Conclusions

The present study demonstrated that CA6 and CA12 exerted acute anticonvulsant effects, but only in the 6 Hz-induced psychomotor seizure test in mice. Since MCT KD has demonstrated evidence of clinical success in the treatment of drug-resistant epilepsy, increasing ketosis by CA6 or CA8 and metformin administered concomitantly may provide some additive effects and reduce overall seizure susceptibility in tested animals. Such an effect was not observed in this study. Instead of this, the results presented here show, for the first time, the proconvulsive effects of the acute co-administration of CA8 and metformin. Caution should be taken when extrapolating our results to humans, as the response to the co-administration of these substances may differ between mice and humans. Further detailed studies are needed to assess the benefit/risk ratio of MCT KD therapy in patients with epilepsy/diabetes who could use it with metformin.

## Figures and Tables

**Table 1:** Effect of CA6 and CA12 on skeletal muscular strength and motor coordination in mice.

Treatment (mmol/kg)	Impairment of Motor Performance (%)	Neuromuscular Strength (mN/g)
control	0	31.1 ± 1.3
CA6 (3)	0	30.3 ± 0.8
CA6 (10)	0	30.5 ± 0.9
CA6 (30)	30	18.4 ± 4.6 **
CA12 (3)	0	30.9 ± 0.7
CA12 (10)	0	29.5 ± 0.5
CA12 (30)	0	27.9 ± 1.5

Results are presented as a percentage of animals showing motor coordination impairment in the chimney test, and as a mean and SEM of grip-strength in millinewtons per gram of mouse body weight (mN/g) from the grip-strength test, assessing skeletal muscular strength in mice, with *n* = 10 mice for each experimental group. The results from the grip-strength test were analyzed using a one-way ANOVA test and Tukey’s post hoc test. Fisher’s exact probability test was used to analyze the results from the chimney test. ** *p* < 0.01 as compared to the control group.

**Table 2:** Effect of metformin treatment on skeletal muscular strength and motor coordination in mice.

Treatment (mg/kg)	Impairment of Motor Performance (%)	Neuromuscular Strength (mN/g)
control	0	36.3 ± 1.6
metformin (100)	0	33.1 ± 1.2
metformin (200)	0	36.6 ± 1.6
metformin (400)	0	34.1 ± 1.0
metformin (600)	0	33.1 ± 0.9

Results are presented as a percentage of animals showing motor coordination impairment in the chimney test, and as a mean and SEM of grip-strength in millinewtons per gram of mouse body weight (mN/g) from the grip-strength test, assessing skeletal muscular strength in mice, with *n* = 10 mice for each experimental group. The results from the grip-strength test were analyzed using a one-way ANOVA test and Tukey’s post hoc test. Fisher’s exact probability test was used to analyze the results from the chimney test.

**Table 3:** Effect of acute and chronic treatment with metformin (100 mg/kg), CA6 (5 mmol/kg)/CA8 (5 mmol/kg) or their combination as well as VPA (150 mg/kg) on skeletal muscular strength and motor coordination in mice.

Treatment	Impairment of Motor Performance (%)	Neuromuscular Strength (mN/g)
*Acute treatment*		
control	0	36.0 ± 1.8
metformin (100 mg/kg)	10	33.0 ± 1.2
CA6 (5 mmol/kg)	10	31.0 ± 1.1
CA8 (5mmol/kg)	0	32.0 ± 1.9
metformin (100 mg/kg) + CA6 (5 mmol/kg)	0	35.7 ± 1.3
metformin (100 mg/kg) + CA8 (5 mmol/kg)	10	31.8 ± 1.3
VPA (150 mg/kg)	0	33.6 ± 1.4
*Chronic treatment*		
control	0	38.0 ± 1.4
metformin (100 mg/kg)	20	37.5 ± 1.5
CA6 (5 mmol/kg)	0	36.5 ± 1.0
CA8 (5mmol/kg)	0	36.9 ± 2.3
metformin (100 mg/kg) + CA6 (5 mmol/kg)	10	35.4 ± 1.5
metformin (100 mg/kg) + CA8 (5 mmol/kg)	0	35.2 ± 1.7
VPA (150 mg/kg)	0	36.6 ± 2.2

Results are presented as a percentage of animals showing motor coordination impairment in the chimney test, and as mean and SEM grip-strength in millinewtons per gram of mouse body weight (mN/g) from the grip-strength test. *n* = 10 mice for each experimental group. The results from the grip-strength test were analyzed using one-way ANOVA test and Tukey’s post hoc test. Fisher’s exact probability test was used to analyze the results from the chimney test.

**Table 4:** Effects of chronic treatment with metformin (100 mg/kg), CA6 (5 mmol/kg)/CA8 (5 mmol/kg) or their combination as well as VPA (150 mg/kg) on mortality in each experimental group after 14 days of treatment.

Treatment Group	Mortality
control	0/30
metformin (100 mg/kg)	0/30
CA6 (5 mmol/kg)	2/30
CA8 (5 mmol/kg)	0/30
metformin (100 mg/kg) + CA6 (5 mmol/kg)	8/30
metformin (100 mg/kg) + CA8 (5 mmol/kg)	0/30
VPA (150 mg/kg)	0/30

The results are presented as the mortality of the mice in response to treatment. Fisher’s exact probability test was used to analyze the results from the experiment.

## Data Availability

The data that support the findings of this study are available from the corresponding author upon reasonable request.

## Sample Availability

Samples of the compounds are not available from the authors.
